# Molecular Detection of Epstein - Barr virus in Nasopharyngeal Carcinoma among Sudanese population

**DOI:** 10.1186/s13027-016-0104-7

**Published:** 2016-11-08

**Authors:** Ali Edreis, Mona Ali Mohamed, Nouh S. Mohamed, Emmanuel E. Siddig

**Affiliations:** 1 Department of Histopathology and Cytology, Faculty of Medical Laboratory Sciences, University of Khartoum, Khartoum, Sudan; 2 Department of Parasitology and Medical Entomology, Faculty of Medical Laboratory Sciences, Nile College, Khartoum, Sudan

**Keywords:** Nasopharyngeal carcinoma, EBV, Cancer

## Abstract

**Background:**

Nasopharyngeal carcinoma (NPC) is the most common cancer arising from the nasopharynx that varies significantly from other cancers of the head and neck in its occurrence, causes, clinical behavior, and treatment. NPC caused by an interaction between infection with EBV and environmental and genetic factors, encompasses a multistep oncogenic process. The frequency of Epstein-Barr virus EBV among nasopharyngeal carcinoma is well known worldwide, however, in the Sudan there is barely a published data. The aim of this study was to detect Epstein-Barr virus (EBV) in nasopharyngeal carcinoma (NPC) biopsies obtained from Sudanese patients using Polymerase Chain reaction.

**Methods:**

This is a descriptive, retrospective hospital based study, conducted at the National Center for ENT diseases and the Faculty of Medical Laboratory Science, University of Khartoum, Khartoum City, Sudan. Archival blocks were obtained from 82 patients diagnosed as having nasopharyngeal carcinoma were molecularly examined for the presence of Epstein-Barr virus.

**Results:**

Eighty two Paraffin fixed tissue sections were examined for the presence of the virus using PCR, EBV was identified in 51/ 82 (62.2 %) samples and couldn’t be identified in 31/ 82 (37.8 %) tissue samples. Out of the 51 infected samples, 33/51 (64.7 %) were found among males and 18/27 (66.7 %) were found among females.

**Conclusion:**

The present study is providing strong evidence supporting the general association of EBV infection in NPC among Sudanese patients.

## Background

Nasopharyngeal carcinoma (NPC) is an epithelial tumor that inflicts the surface of the nasopharynx. NPC was firstly described and coined as a separate entity thanks to Regaud and Schmincke, [1, 2]. Importantly, According to World Health Organization (WHO), NPC encompasses squamous cell carcinoma (Type I), non-keratinizing carcinoma (Type II) and undifferentiated carcinoma (Type III) [3, 4]. Outstandingly, NPC is infrequent in the United States and many other countries, representing less than 1 case per 100,000 in most populations; exceptionally it is common in southern regions of China [5]. Furthermore, in United Kingdom the incidence of NPC is 0.25 per million at age 0–14 years old, and 0.8 per million at age 10–14 years old (Brennan, 2006). Additionally, it affects Arabs in North Africa as well as Eskimos in Antarctica [6]. Significantly, Sudan is considered an intermediate area of endemicity for this type of cancer [7, 8]. In Sudan, due to paucity of data and registry, the only epidemiological available report return to 1983 in which, 374 cases of NPC were recorded in the Sudan Cancer Registry (SCR) and 512 cases were seen at the Radiation and Isotope Centre, Khartoum (RICK). NPC formed 5.8 % of all cancer cases in the SCR and 7.2 % at the RICK [9].

NPC seems to occur due to a multifactorial process as well as investable corollary in which it involved the contributions of *Epstein Barr* Virus (EBV), ethnic background, and environmental carcinogens. All of these seem to play an important role in the development of NPC. Epstein-Barr virus (EBV) is a canonical human herpes virus that is associated with several malignancies, namely, Burkitt lymphoma (BL), Hodgkin lymphoma (HL), gastric cancer, and, most importantly, nasopharyngeal carcinoma (NPC) [7]. Intriguingly, in all these tumors, EBV infection is predominantly latent. Inlatent infection, expressed EBV genes are restricted to six EBV nuclear antigens (EBNA1, −2, −3A, −3B, −3C, and -LP), three latent membrane proteins (LMP1, −2A, −and 2B), and two small non polyadenylated RNAs (EBER1 and −2).

LMP1, a constitutive member of the tumor necrosis factor receptor super family, moreover, is considered to be a potent oncogenic protein owing to of its transforming effects on rodent fibroblasts as much as it is essential for the immortalization of B lymphocytes along with epithelial cells, prominently, this protein is highly expressed in most EBV-associated human malignancies [10, 11].

The aim of the present study was to screen for EBV among Sudanese NPC patients.

## Methods

### Study design

This was a descriptive, retrospective; hospital - based study, conducted at nasopharyngeal carcinoma clinic within the ENT Khartoum Teaching Hospital, and the Faculty of Medical Laboratory Science, University of Khartoum, Khartoum City, Sudan. during the period of October 2015 till January 2016. In this manner archival blocks of 82 patients diagnosed with nasopharyngeal carcinoma were retrieved for the detection of EBV.

### Preparation of Formalin fixed Paraffin block prior DNA extraction

Formalin fixed, Paraffin-embedded blocks were cut into 12 μm thick sections using rotary microtome (Leica), and the tissue transferred into eppendorf tube under stringent sterile condition to avoid contaminations (by using a sterile knife for each block, and after each block the block holder and knife holder were washed using 70 % ethanol). After that For each eppendorf tube 1 ml xylene was added twice over for 10 min to dissolve the paraffin wax from the tissue, and then rehydrated through a series of ethanol washes starting with 95 % followed by 90 %, and 70 % for 3 min for each. Proteins were digested by proteinase K. Buffer containing denaturing agent (sodium dodecyl sulfate (SDS)), was added to facilitate digestion as described by Pikor and his associates [12]. Nucleic acids were purified from the tissue lysate using buffer saturated phenol with the aid of high speed centrifugation (14.000 rpm). Ensuing phenol extractions, RNase A (at 100 μg/ml for 1 h at 37 °C) was added to eliminate contaminating RNA. Additional phenol extractions following the incubation with RNase A were used to remove any residual enzyme. 3 M Sodium acetate and 100 % isopropanol were added to precipitate DNA, subsequently, high speed centrifugation (14.000 rpm / 10 mins) was used to pellet the DNA and ease removal of isopropanol. Washing with 70 % ethanol was performed to dispose of excess salts, followed by centrifugation to re-pellet the DNA as described by Pikor and his associates [13, 14]. DNA is resuspended in distilled water, quantified and stored at −20 °C. Purified DNA was afterwards used in downstream applications of PCR.

### Assessment of DNA Quality

DNA quality was assessed using 1 % Agarose gel electrophoresis and further evaluated in terms of the A260/280 ratio in a Nanodrop 1000 apparatus.

### Molecular identification by polymerase chain reaction

We used primer for the EBV-LMP-1 gene forward 5́- CCG AAG AGG TTG AAA ACA AA-3 and Reverse 5-GTG GGG GTC GTC ATC ATC TC-3.

### Molecular identification by polymerase chain reaction

Polymerase chain reaction (PCR) was carried out for amplification of target EBV genome by using genomic DNA template (1.5 μL of EBV detection). The PCR was performed in one step (single tube) in a 25 μl final volume using iNtRON’s Maxime PCR PreMix Kit (i-Taq) (Korea) according to manufacturer instruction. Then the samples were pre-incubated at 110 °C for 4 min, followed by initial denaturation at 95 °C for 5 min, and 35 cycles of denaturation at 94 °C for 1 min, and 35 cycles of annealing at 60 °C for 1 min and 35 cycles of elongation at 72 °C for 1 min, and final elongation at 72 °C for 10 min, performed on a thermocycler (SensoQuest brand).

### Statistical analysis

The statistical analysis of the results was done using the SPSS (vs. 16.0) statistical software. The Chi-Squared test was used to compare the frequencies of the categorical variables. A value of *p* < 0.05 was considered statistically significant.

## Results

The age of the patients was ranged between 10 and 87 years and the median age was 43 years. The distribution by gender was found to be 55 (67.1 %) males and 27 (32.9 %) females with male/female ratio 2.03 to 1.00. Regarding WHO Histological classification, twenty (24.4 %) and 62 (75.6 %) of the biopsies were classified as types II, III respectively; cases of type I Nasopharyngeal carcinoma were not available (Table 1). The distribution of patients by residence, noticeably, most cases of NPC were coming from Western Sudan representing 22/82 (25.6 %) followed by Southern Sudan constituting 18/82 (22 %), as shown in (Table 1).

**Table. 1:** Shows the distribution of study population by gender, age, residence and histopathology

Characteristics	
Age range	10–87
Gender	
Male	55 (67.1 %)
Female	27 (32.9 %)
Histology	
WHO I	0 (0 %)
WHO II	62 (75.6 %)
WHO III	20 (24.4 %)
Residence	
North	15
South	18
West	21
East	11
Central	17

Among 82 NPC tissue specimens, EBV DNA was identified in 51/ 82 (62.2 %) samples and failed to be identified in 31/ 82 (37.8 %) tissue samples. Among the 51 infected samples, Viral positivity was expressed in 33/51 (64.7 %) among males, whereas 18/27 (66.7 %) was expressed among females (Table 2).

**Table. 2:** Shows Distribution of the study population by gender and EBV infection

Gender	EBV (LMP 1)		Total
	Negative	Positive	
Male	22 (40 %)	33 (60 %)	55
Female	9 (33.3 %)	18 (66.6 %)	27
Total	31 (37.8 %)	51 (62.1 %)	82

The highest frequency of infection rates were seen among age group 21–40 years representing 16/51 (31.3 %) ensued by ages ranged between 41–60 and 61–80 years, constituting 14/51 (27.4 %) correspondingly, then 0–20 years and 80+ representing 4/51 (7.8 %) and3/51 (5.9 %) respectively (Table 3).

**Table. 3:** Distribution of the study population by age and EBV infection

Age Group	EBV (LMP 1)		Total
	Negative	Positive	
0–20	3	4	7
21–40	9	16	25
41–60	15	14	29
61–80	4	14	18
More than 80	0	3	3
Total	31	51	82

With regard to EBV infection by residence, notably, by far the majority of infections were detected among Western region’s populations, representing 15/ 51 (29.4 %) followed by Southern region’s populations, constituting 12/51 (23.5 %) as shown in (Table 4).

**Table. 4:** Distribution of the study population by residence and EBV infection

Residence	EBV (LMP 1)		Total
	Negative	Positive	
Central	6	11	17
Northern	10	5	15
Southern	6	12	18
Eastern	3	8	11
Western	6	15	21
Total	31	51	82

## Discussion

Nasopharyngeal carcinoma (NPC) is relatively a rare cancer in most parts of the world, although, it is the most commonly diagnosed head and neck cancer in certain region of Southeast Asia. Evolving body of researches indicates that the age standardized incidence rate is less than 1 per 100.000 people per year for either gender worldwide [15, 16]. Isolated northern populations such as Eskimos and Greenlanders also show high incidence. There is a moderate incidence in North Africa, Israel, Kuwait, the Sudan and parts of Kenya and Uganda. Furthermore, in most populations, the age standardized annual incidence rate of NPC is much higher among males compared to females, with male to female ratio around 2–3: 1 in high and moderate risk areas.

Infection with EBV has been implicated in the development of nasopharyngeal carcinoma by several different lines of compelling evidence. Historically, the first indication was the observation that sera from African and American patients with nasopharyngeal carcinoma were often more positive for precipitating antibodies to antigens prepared from cultured Burkitt lymphoma cells than controls [17]. This observation has since been confirmed in serological studies showing elevated titers of IgG and in particular IgA antibodies against EBV viral capsid, early and nuclear antigens in nasopharyngeal carcinoma patients data being less convincing for type I than types II and III, manifesting as apparent ethnic variations [18]. Much compelling than the sero-prevalence surveys in patients are, the results of a prospective study of 9699 persons which showed that presence of IgA anti-EBV viral capsid antigen antibodies or neutralizing EBV specific anti-DNase antibodies correlated with subsequent risk for nasopharyngeal carcinoma [19].

In the current study, the correlation between NPC and the presence of the EBV genome was evaluated. An extremely significant positive correlation was found based on two genes encoding for EBV viral proteins. Exhaustive studies have investigated this correlation; nevertheless, these findings are greatly contradictory. The role of EBV infection in the etiology of NPC in Sudan had been reported since 1979 [20]. In addition, a recent study used 53 biopsies obtained from Sudanese patients 43 with NPC beside ten normal samples were used to investigate the presence of viral genome in non malignant cells; the study was aimed at investigating the presence of EBV using EBER-ISH. Intriguingly, their results showed that all nasopharyngeal carcinoma biopsies (100 %) were positive for EBER1among all carcinoma cells, however, no hybridization was observed among all 10 nonmalignant tissues [21]. Surprisingly, these are findings greatly discordant from the prevalence of EBV obtained in our present study (62.2 %), possibly this might be attributed to their small sample size 43 compared to ours which is 82 specimens. Furthermore, a study was conducted by Ahmed and associates [9], on 150 biopsies obtained from Sudanese patients with NPC, was screened for the presence of EBV genes; EBV nuclear antigen-4 (EBNA-4) and latent membrane protein-1 (LMP1) using Polymerase Chain Reaction (PCR) [25]. Significantly, EBV genes were detected in 92/150 (61.3 %) tissue samples, accordingly, these results are in agreement with our prevalence (62.2 %). Interestingly, the incidence of EBV among South Africa patients with NPC were much higher, in a study conducted by Janse and his associates, they investigated the incidence of EBV among 38 NPC Patients using PCR to amplified EBER region, their results shows that EBV were detected in 82 % (31/38) of the tumours [21].

The distribution of NPC by gender, Among 82 NPC cases, there were 55 (67.1 %), males and there were 27 (32.9 %) females with male/female ratio 2.03 to 1.00, this indicating that the number of males with NPC was much higher than females, growing body of researches have previously reported this finding [7, 8, 21].

Strangely enough, the frequency of EBV infection was higher among males, however, when comparing the per centage of EBV infection within each group, (66.7 %) of women were relatively infected whereas (64.7 %) were men. Moreover, meaningful evidences have shown that NPC is much often diagnosed among men compared to women, interestingly, it tends to occur at an earlier age than do most cancers [22–25]. However, there are no studies, specifically, focusing on the prognostic impact of gender on NPC. Moreover the EBV positive cases were reported to be occurring between 2^th^ and 4^th^ decades of life, followed by 4^th^ to 6^th^and 6^th^ to 8^th^ decades of life, our results in are in consistent manner resembling previous reports [9].

Regarding the histopathological subtypes of NPC among our current study populations, we found them to be WHO type II and III, whereupon in accordance to report from other endemic areas [26]. Additionally, our results are in accord with previous studies reported in Sudan [8]. Interestingly, these subtypes i.e. II and III are associated with high rates of EBV detection [27].

In regard to the distribution of EBV infection by residence and, the great majority of infections were detected among Western region’s populations, representing 15/ 51 (29.4 %) followed by fully-fledged Southern region’s populations, constituting 12/51 (23.5 %), these finding are in concordance to that recently reported by Ahmed and colleagues [9], which show a high incidence of EBV among western region’s populations. Additionally, a groundbreaking study conducted by Abdullah and his fellows, reported a high frequency of NPC in western Sudan, however, they attributed their data to the presence of radioactive uranium in areas [8].

## Conclusion

In conclusion, the data presented in this study suggest that possibly EBV could be involved in initiating NPC among Sudanese Patients. Using EBV as an excellent marker might pay off for NPC especially for people with a family history of NPC.

## Abbreviation

EBNA: *Epstein Barr* nuclear antigen
EBV: *Epstein Barr virus*
LMP: Latent membrane protein
NPC: Nasopharyngeal carcinoma
PCR: Polymerase chain reaction
