# Erratum to: Molecular Detection of Epstein - Barr virus in Nasopharyngeal Carcinoma among Sudanese population

**DOI:** 10.1186/s13027-016-0108-3

**Published:** 2016-11-30

**Authors:** Ali Edris, Mona Ali Mohamed, Nouh S. Mohamed, Emmanuel E. Siddig

**Affiliations:** 1 Department of Histopathology and Cytology, Faculty of Medical Laboratory Sciences, University of Khartoum, Khartoum, Sudan; 2 Department of Parasitology and Medical Entomology, Faculty of Medical Laboratory Sciences, Nile College, Khartoum, Sudan

After publication of this article [1], the authors noticed Ali Edris’s name was misspelled as Ali Edreis. The correct spelling of the author’s name is included in the author list of this erratum and has been updated in the original article [1].
